# School Locations and Traffic Emissions — Environmental (In)Justice Findings Using a New Screening Method

**DOI:** 10.3390/ijerph120202009

**Published:** 2015-02-11

**Authors:** Philine Gaffron, Deb Niemeier

**Affiliations:** 1 Institute for Transport Planning and Logistics, Hamburg University of Technology, 21071 Hamburg, Germany; 2 Department of Civil and Environmental Engineering, University of California Davis, One Shields Avenue, Davis, CA 95616, USA; E-Mail: dniemeier@ucdavis.edu

**Keywords:** school siting, children’s health, transport emissions, PM_2.5_, road traffic, screening, environmental justice, California

## Abstract

It has been shown that the location of schools near heavily trafficked roads can have detrimental effects on the health of children attending those schools. It is therefore desirable to screen both existing school locations and potential new school sites to assess either the need for remedial measures or suitability for the intended use. Current screening tools and public guidance on school siting are either too coarse in their spatial resolution for assessing individual sites or are highly resource intensive in their execution (e.g., through dispersion modeling). We propose a new method to help bridge the gap between these two approaches. Using this method, we also examine the public K-12 schools in the Sacramento Area Council of Governments Region, California (USA) from an environmental justice perspective. We find that PM_2.5_ emissions from road traffic affecting a school site are significantly positively correlated with the following metrics: percent share of Black, Hispanic and multi-ethnic students, percent share of students eligible for subsidized meals. The emissions metric correlates negatively with the schools’ Academic Performance Index, the share of White students and average parental education levels. Our PM_2.5_ metric also correlates with the traffic related, census tract level screening indicators from the California Communities Environmental Health Screening Tool and the tool’s tract level rate of asthma related emergency department visits.

## 1. Introduction

As McDonald aptly noted [1], schools are a “long-lived and spatially fixed infrastructure” (p. 184). As such, factors key to successful school siting have been given considerable thought over the years, which is fully evident in the plethora of U.S. state and local guidance on the planning, construction and renovation of new and existing schools (e.g., see [2]). Yet, the voluntary nature of most of the available guidance can diminish the need for critical assessment of school locations, existing or planned. Despite the existence of a now significant body of evidence that links a number of important health issues in children to the locational aspects associated with a school, more than 20 U.S. states lack legislation ensuring that schools will not be built near manmade hazards, and only 10 states prohibit school siting near health hazards [3]. The latter group includes California, where Senate Bill 352 [4] was passed in 2003 to prevent new schools from being sited “within 500 feet from the edge of the closest traffic lane of a freeway or other busy traffic corridor” (ibid., preamble) among other hazardous activities. 

However, no guidance or legislation relating to new schools creates any context for the assessment of existing school sites. In addition, the cost of performing detailed site analyses is often a key limitation faced by local administrations, even if they do wish to provide a healthy environment for their school children. Because of this, screening processes have been developed to identify key health hazards—alone and in combination—such as toxic waste sites, potential chemical exposure pathways, and more recently the implications for air quality resulting from road proximity. These processes are intended to help local decision makers in assessing the level of affectedness of different communities, the need for potential mitigation and the suitability of certain areas for siting either new sensitive or hazardous uses. As we will show, many of the screening methods or indicators developed to date rely on indirect measures of air quality and are not sufficiently finely resolved to assess individual new or existing school sites. In this paper, we present a robust, new screening method that allows a site specific examination of air pollutant effects near roadways.

## 2. Children, Schools, Traffic and Health 

The built environment influences children’s health across two critical dimensions related to the locational aspects of schools: the primary modes of travel to and from school and the proximity and density of traffic. Scholars have shown that urban form plays an important role in how children travel to and from schools [5,6], and that with the growth of suburbia, not only has children’s physical activity declined in general [7] but travel to schools has also shifted dramatically from non-motorized modes of travel to cars [8,9]. This shift in activity patterns as a result of changes in the built environment has been associated with higher levels of school age obesity [10]. Additionally, in most urbanized and some suburban areas, concerns about traffic and safety have also increased over time, further solidifying auto-based travel choices, particularly for children. 

Higher traffic density has been linked not only to the development of obesity [11], but also to adverse respiratory effects. The prevalence of respiratory disease is higher for children living closer to high volume roadways [12,13,14], with decreasing lung function associated with higher traffic intensity, particularly around schools [15]. Findings also show that levels of air pollutant concentrations at downwind schools are significantly correlated with distance from high volume roadways and increasing truck traffic [16]. The actual exposure effects of high concentrations of air pollutants are a function of both indoor and outdoor air quality and the time spent in the various microenvironments, but evidence suggests that as much as 29% of indoor air concentrations are related to outdoor sources [17]. Childhood asthma is already the most common chronic respiratory disease in children [18] and these children are also less likely to walk to schools, relying instead on bus or vehicle travel [19], which potentially further exacerbates obesity concerns.

States have been fairly aggressive in identifying and developing guidance that addresses the need for schools that can be accessed by walking or biking. One key program in this effort has been the Safe Routes to School (SRS) Program, which was included as part of the SAFETEA-LU (05–09) legislation. The SRS program has provided funding for capital infrastructure designed to improve non-motorized travel to local schools. States have been less assertive in establishing clear guidance around school siting with respect to air quality-related health concerns, and the U.S. Environmental Protection Agency (EPA) has taken the lead, recently releasing voluntary guidance aimed at integrating environmental considerations into school siting decisions. Particular attention has been paid to pollutant exposure, the availability of alternative transportation modes, energy efficiency and dual use as an emergency shelter [20]. There have been some criticisms of EPA’s guidance, though, particularly for its reliance on reducing, rather than avoiding the effects of environmental hazards and its lack of awareness around racial segregation issues [21].

There is a significant body of literature that suggests that air pollution burdens fall disproportionately on minority communities, e.g., [22,23,24]. Also, schools with higher levels of air pollutants or nearer busy roads have been shown to have higher proportions of poor students and students of color [25], many of whom also suffer from proportionally higher respiratory risks and show evidence of lower academic performance, even after controlling for school, family, and geographic factors [26,27]. State guidance is limited, though, with only 14 states specifically restricting school siting at or near major sources of air pollution [28]. Other states offer school siting guidance similar to EPA’s policy, focusing on reduction rather than avoidance. Even if employed for the purposes of reducing air pollutant exposure, the cost of fully implementing EPA’s air pollution analysis guidelines would likely be time and cost prohibitive for most local school districts. EPA suggests running dispersion models [20], which require a high level of technical expertise and considerable time input to set up, to better assess air quality.

A number of screening programs have been developed as a first step, though, particularly in California, which are designed to help identify clear cases where air pollution is likely to be a problem. Although not specifically developed to evaluate air pollution at schools, the Air Resources Board (ARB) has used a modified version of the community-based screening method developed by Sadd, Pastor, Morello-Frosh, *et al.* [29]. The ARB analysis method identifies those communities that are likely to be most highly impacted by air pollution by combining data on air pollution exposure with indicators of low income status [30]. However, this method relies on air pollution monitor data, rather than estimating emissions from close proximity major sources (e.g., high volume roadways). This limits the application to site specific evaluations such as that of schools. The California Environmental Protection Agency (CEPA) has also developed a screening tool intended to help decision makers and communities to assess the environmental health vulnerability of Californian census tract populations, Cal Enviro Screen 2.0 or CES. It incorporates information on a range of pollutants (e.g., ozone and PM_2.5_ concentrations, amount of pesticides used per areal unit and road traffic densities), population characteristics (e.g., percent of population under age 10 and over age 65, percent living below twice the federal poverty level) and health outcomes such as incidences of asthma and low birth weight [31]. As with ARB’s screening method, the air pollution indicators are partly based on data from monitoring stations. Both these and the traffic load related indicators are interpolated to tract level values which results in the same limitations when attempting to use the information to assess individual school sites.

The purpose of this paper is to demonstrate a robust method that can cost-efficiently help to screen out potentially poor school sites. The method is also useful for prioritizing schools for renovation and/or implementation of improved school ventilation systems. Using the Sacramento, California region, we show that our method performs better than the current methods available in California and helps bridge the gap between less robust screening methods and the more accurate, but resource intensive dispersion modeling.

## 3. Empirical Setting

California has one the largest inventories of public schools in the country: almost 10,000 public schools serving about 6 million students [32]. It is estimated that 71% of the classrooms are at least 25 years old, and of those 30% are at least 50 years old and 10% are at least 70 years old, so although regulations exist to guide the siting of new schools, e.g., [4], attention has now turned to the infrastructure needs of existing schools. It is estimated that upwards of $100 billion is needed to address deferred maintenance, seismic improvements, modernization and new construction needs. The majority of funds have been identified for health, safety and disability concerns [32]. Identifying those schools most likely to be under conditions of high air pollutant exposure for children would help to prioritize future rehabilitation and renovation efforts. 

Green *et al.* have shown in 2004 that California public K-12 schools with higher proportions of Black and Hispanic students as well as those from low income families are more likely to be located near busy roads than are schools with student bodies containing lower proportions of these groups [25]. However, their study used point data of school addresses and the busiest network link within 150m of those points for classifying schools into four traffic level groups. While this provided a good impression of the overall picture in California, the approach does not take into account different vehicles types and speeds. It also does not quantify the cumulative emissions that vehicles travelling on different network links near schools may produce and it does not take account of the fact that school sites are not points. The affected properties can have very large or very small portions located near the busiest roads in their proximity which will influence actual exposure levels of the students. 

Our study examines actual school properties in the six-county Sacramento Area Council of Governments (SACOG) region and uses modeled traffic loads from all roads within 150 m of the school parcel to quantify the per area emissions affecting each school.

## 4. Methods

### 4.1. Schools Data 

Schools were identified using a GIS point shape provided by the SACOG GIS Mapping Center and a land-use parcel polygon shape from SACOG’s 2008 land-use model I-PLACE^3^S (www.sacog.org/mapping/clearinghouse/). From these sources, 1126 schools were identified, from which we selected the 553 public K-12 schools that could be identified as a point location within an I-PLACE^3^S land-use parcel designated as “K-12 Schools” or a differently designated parcel that could manually be verified as a school location. Finally, we kept only those schools located in parcels that intersected with a 150 m buffer of a roadway as identified in the traffic loaded network from the SACOG travel demand model. The buffer distance of 150 m was chosen to mirror the distance at which traffic density was assumed to have an effect in the CES (Table 1). This distance also coincides with that at which a review by Karner *et al.* [33] found ultrafine particle numbers drop to 50% of their roadway edge concentration level.

Data on socio-economic characteristics of the student bodies were obtained from the California Department for Education in the form of the Academic Performance Index (API) data files for 2010 [34]. Using the 14-digit school code found in both the points shape and the API dataset, the information on student bodies was joined to the school locations. The API dataset includes the number of students enrolled in 2010, the school’s Academic Performance Index 2010, the average parental education level, and the percentage of the student body by Black/African American, Asian, Hispanic/Latino, White and two or more races. The data also include the percentages of students eligible for free or reduced price meals and those designated as English learners. The figures on enrollment differed from and were generally lower than those provided by SACOG for the same schools and the same year. Since the remaining socio-economic data was also taken from the API dataset, it was decided to retain these enrollment figures for consistency.

There was more than one school located within a parcel for 38 of the parcels identified in the land-use data. As might be expected, the students’ socio-economic characteristics and the school’s academic performance for two schools within the same parcel were highly correlated (for Pearson’s *r*: *p* > 0.05 in all 38 parcels, Table 2) Note that three parcels contained more than two schools (2 × 3, 1 × 5). For the correlation analysis the five additional schools were disregarded. For both single school parcels and multiple school parcels, the emission loads were allocated at the parcel level. Instead of using individual school data for schools sharing a parcel, we used mean values weighted by enrollment numbers for the characteristics of the students in these parcels so that the final dataset contained 510 data points, one for each school parcel.

### 4.2. Emissions Data 

To calculate the emission loads to be assigned to the school locations, we used the 2008 traffic-loaded network from the regional travel demand model, SACSIM, and the California Air Resources Board’s (CARB) Emission Factor (EMFAC) Model 2011. The SACSIM network contains the length in miles for each roadway, the capacity class, the modeled volume (one way)—*i.e.*, the average annual daily traffic (AADT) for that link-, and the congested speed (calculated from the modeled travel time on each link and its length, often equivalent to the free flow speed). Where two unidirectional model network links that represented bidirectional parts of the actual road network were spatially congruent, they were combined into a single link for which the modeled volumes were added and the congested speeds represented as a weighted average. Functional links in the modeled network with no equivalent in the real world (e.g. collector links summarizing traffic to and from residential blocks) were removed.

**Table 1:** CES variables included in this study (based on [31]).

Variable	Definition
PM_2.5_	Annual mean concentration of PM_2.5_ (average of quarterly means), over three years (2009–2011) spatially extrapolated from air quality monitoring network data (μg/m^3^)
Diesel particulate matter	Spatial distribution of gridded diesel PM emissions (4 × 4 km) from on-road and non-road sources for a 2010 summer day in July (kg/day)
Traffic density	Sum of 2004 traffic volumes adjusted by road segment length (vehicle-km per hour) divided by total road length (km) within 150 m of the census tract boundary (no unit)
Asthma *	Spatially modeled, age-adjusted rate of emergency department (ED) visits for asthma per 10,000 inhabitants of tract (averaged over 2007–2009; no unit)
Low birth weight *	Percent infants born with low birth weight (<2500 grams), spatially modeled (averaged over 2006–2009)

* Spatially allocated according to home address of patient/mother respectively.

**Table 2:** Correlation between student characteristics of largest school (by enrollment) *vs.* smaller schools in parcels with two schools; N = 38 for each group.

Variable	Pearson’s *r*	Significance
Academic Performance Index 2010	0.562	0.002
% of Black/African American students	0.733	0.000
% of Asian Students	0.399	0.019
% of Hispanic/Latino Students	0.671	0.000
% of White Students	0.667	0.000
% of Students belonging to Two or More Races	0.344	0.035
% of Students Eligible for Free/Reduced Price Meals	0.524	0.001
% of Students Designated as English Learners	0.487	0.002
School-Wide Average Parental Education Level	0.666	0.000

Note: For descriptive statistics of overall samples and more information on the variables (Table A1 in the Appendix).

The SACSIM model output is available for four different time periods, morning 7:00–10:00, midday 10:00–15:00, afternoon 15:00–18:00 and evening/night 18:00–7:00. Since we were interested in the emissions that students would be exposed to while at school, we used the morning and midday network data for our calculations. 

EMFAC provides emission rates for carbon monoxide (CO), nitrous oxides (NO_x_) and particulate matter (PM_10_ and PM_2.5_) among others. We used PM_2.5_ for three reasons: first, its strong correlation with respiratory illnesses (e.g., [35,36,37]) second, the fact that there if no known evidence for a no-effects threshold for this pollutant ([37,38]) and third, because the Sacramento region is in non-attainment for the federal PM_2.5_ standards [39]. EMFAC can be used to estimate the average annual emission values in grams per vehicle mile for different vehicle classes using different fuel types (diesel/gasoline) in 5 m per hour (mph) speed bins covering a range from 5 to 70 mph. Along with the emission rates, EMFAC also provides data on the average miles per day covered by each vehicle class. 

From this information, we calculated an average emission rate per vehicle mile as generated by the entire fleet in each speed bin (the traffic-loaded network produced by SACSIM does not distinguish by vehicle types). To allocate the emission rates to the modeled link loads, the emission rates for individual mph speeds were extrapolated from the speed bin results. The EMFAC emissions rates are also estimated by county and air basin using region specific meteorology. The six counties in the SACOG region intersect with three different air basins, Lake Tahoe, Mountain Counties and Sacramento Valley. Since the SACSIM roadway network does not include roads in the Lake Tahoe air basin, only the latter two were taken into account. Emission rates were then allocated to the roadway network in accordance with each roads location within a county and one of the two air basins.

For a given speed bin, the PM_2.5_ load is calculated by multiplying the vehicle miles travelled (VMT) for each vehicle class by the relevant emission factor. The vehicle class results are summed for total emissions and divided by total VMT to get emissions/vehicle mile:

(1) $$\frac{\sum (VMT_{class,speed}\times ef_{class,speed}^{PM2.5})}{\sum VMT_{class,speed}}=PM2.5_{speed}$$

where *VMT_class, speed_* denotes the vehicle miles/day of individual vehicle classes within a given speed bin; $ef_{class,speed}^{PM2.5}$ is the PM_2.5_ emission rate for each vehicle class in the speed bin in g/vehicle mile and *PM_2.5__speed_* is the PM_2.5_ emission factor for each speed bin in g/vehicle mile.

The emission factors for speeds in individual mph between 5 and 70 were extrapolated from the values for the 5mph speed bins and emission factors assigned to each network link in accordance with their congested speed. PM_2.5_ load in g/link mile of SACSIM network was calculated thus:

(2) $$V_{link}\times PM2.5_{speed}\times linklength=PM2.5\;link\;load$$

where V_link_ denotes the congested speed on the given link.

We then buffered each roadway link in the network to 150m on both sides, the area of the buffer was calculated and the PM_2.5_ roadway segment emissions load normalized to the buffer area in g/1000 m^2^. 

### 4.3. Assignment of Emission Loads to Schools

Overlapping buffers were intersected to create contiguous emissions loads from adjacent or intersecting roadway links (the per area contributions of PM_2.5_ from each buffer were added together and the resulting emissions load assigned to the new buffer fragment). The resulting contiguous emissions load buffer shape was intersected with the school parcels, and the per area PM_2.5_ load of each buffer (fragment) that intersected with any school parcel was normalized (if a buffer with x g/1000 m^2^ of PM_2.5_ covered half of a school parcel, the resulting per area load for that parcel is x/2. If another buffer with load y covered a quarter, another y/4 g/1000 m^2^ would be added to give an overall per area load of the parcel of (x/2) + (y/4) g/1000 m^2^) to the area of that parcel and, where multiple buffer (fragments) intersected with a parcel, the contributions were added:

(3) $$\sum_{1}^{n}\left(\frac{area\;buffer\;fragment\;in\;parcel}{area\;parcel}\times PM2.5\;buffer\;load\right)=PM2.5\;parcel\;load$$

where the areas of buffer fragments and parcels are in 1000 m^2^, the buffer and parcel loads in g/1000 m^2^ and *n* is the number of different buffers intersecting a school parcel. Emission loads from both the morning and midday modeling periods were summed for each school location. We use this estimate as our metric of the *cumulative* emission load at the respective school locations.

### 4.4. Environmental Health Screening Variables 

We assigned the tract level CES health outcome and pollution variables to our school dataset by their location to examine correlations with our emissions measure (see Table 1 for variables chosen). We note that the CES variables do not correspond to the same year or time period. We have used these variables because they represent the current information available from the screening tool. Most of these variables are also close to, or chronologically congruent with the reference years of our data (emission loads: 2008, schools data: 2010). Since the health effects of exposure to particulate matter are generally associated with long-term exposure, the different timeframes are not likely to present a problem for our work.

## 5. Analysis

We tested for correlations between the PM_2.5_ emission loads of a school location, the socio-economic characteristics of the student body and the health outcomes quantified for the CES. We also looked at the relationship between the PM_2.5_ metric used in this study and related pollution variables from the CES. The analyses were conducted both for the entire sample and separately for all schools found in incorporated areas and those in unincorporated locations for the following reason: in California, the distinction between incorporated *vs.* unincorporated areas generally describes more *vs.* less densely settled—and thus often more *versus* less trafficked - areas and we did find a significant difference between the mean PM_2.5_ load of school locations in incorporated areas *versus* those in unincorporated areas ($\bar{x_{incorporated}}$ = 0.262 g/1000 m^2^,$\;\bar{x_{unincorporated}}$ = 0.1301 g/1000 m^2^; t = 3.633, *p* < 0.005) (descriptive statistics for the variables can be found in Table A1 in the Appendix). The results of the correlation analysis (Pearson’s *r*) are shown in Table 3. 

We report both the correlation coefficient (*r*) and coefficient of determination (R^2^) to describe shared variance in the variables. Note that schools found in the same location, *i.e.*, occupying the same land-use parcel, have been treated as one data point with enrollment-weighted averages for the relevant variables. The total sample comprised 553 schools with 250,433 students (153,471 schooled in incorporated areas and 96,962 in unincorporated areas).

**Table 3:** Correlation of schools’ PM_2.5_ loads with socio-economic and CES variables (significant relationships are shown in bold type).

Analysis Area	Socio-Economic/Health Variable	*r*	R^2^	Significance
ALL	Academic Performance Index 2010	−0.209	0.044	0.000
INC.		−0.241	0.058	0.000
UNINC.		−0.126	0.016	0.071
ALL	% Black/African American Students	0.264	0.070	0.000
INC.		0.276	0.076	0.000
UNINC.		0.194	0.038	0.004
ALL	% Asian Students	−0.019	0.000	0.667
INC.		−0.105	0.011	0.072
UNINC.		0.119	0.014	0.080
ALL	% Hispanic/Latino Students	0.154	0.024	0.000
INC.		0.120	0.014	0.041
UNINC.		0.153	0.024	0.024
ALL	% White Students	−0.205	0.042	0.000
INC.		−0.168	0.028	0.004
UNINC.		−0.211	0.044	0.002
ALL	% students of Two or More Races/Ethnicities	0.122	0.015	0.006
INC.		0.108	0.012	0.064
UNINC.		0.166	0.027	0.015
ALL	% Students Eligible for Free/Subsidized Meals	0.204	0.042	0.000
INC.		0.200	0.040	0.001
UNINC.		0.226	0.051	0.001
ALL	% Students Designated as English Learners	0.051	0.003	0.251
INC.		−0.023	0.001	0.689
UNINC.		0.191	0.036	0.005
ALL	Average Parental Education Level	−0.178	0.030	0.000
INC.		−0.171	0.029	0.003
UNINC.		−0.207	0.043	0.002
ALL	CES: Annual Mean PM_2.5_ μg/m^3^	0.149	0.022	0.001
INC.		0.146	0.021	0.012
UNINC.		0.182	0.033	0.007
ALL	CES: Particulate Matter from Diesel (kg/day, July)	0.458	0.210	0.000
INC.		0.480	0.230	0.000
UNINC.		0.344	0.118	0.000
ALL	CES: Traffic Density	0.308	0.095	0.000
INC.		0.230	0.053	0.000
UNINC.		0.535	0.286	0.000
ALL	CES: Rate of Asthma Related Emergency Department Visits	0.134	0.018	0.002
INC.		0.117	0.014	0.045
UNINC.		0.225	0.050	0.001
ALL	CES: % of Low Weight Births	0.071	0.005	0.108
INC.		0.078	0.006	0.182
UNINC.		0.034	0.001	0.617

## 6. Findings 

### 6.1. Socio-Economic Variables 

Looking at the entire analysis area, seven out of nine of the socio-economic parameters characterizing the student bodies of public schools in the SACOG region correlate highly significantly with the road traffic related pollution burden of the schools’ locations as expressed in PM_2.5_ g/1000 m^2^ from morning and midday traffic (7.00–15.00 h; see Table 3). For the significantly correlated variable pairs, R^2^ values range between 0.015 and 0.070 (or 1.5%–7.0% shared variance between the variable pairs) and while these are not large in absolute terms, they are nevertheless noteworthy as they describe a relationship between two variables that are not expected to exert a direct influence on each other. 

**Figure 1:**
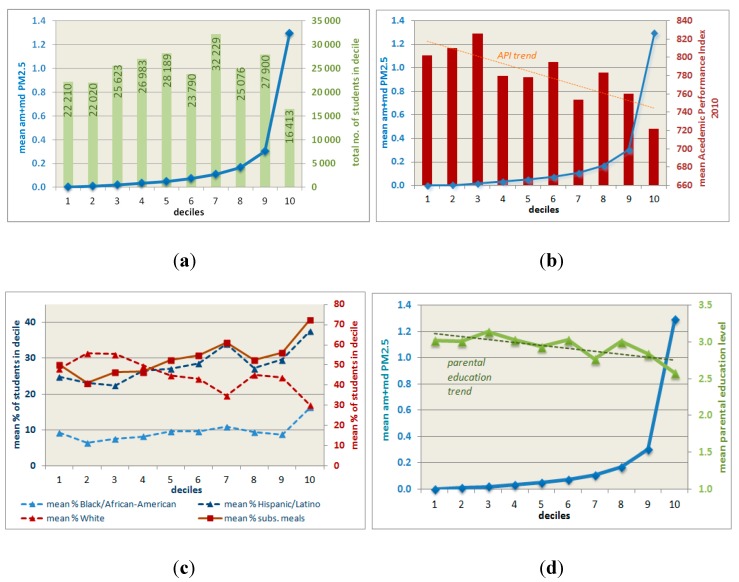
(**a-d**) Diagrams showing means of selected socio-economic variables within deciles of all schools’ am + md PM_2.5_ emission loads (deciles along the x-axes, N = 510).

In the full sample (N = 510), higher PM_2.5_ levels are significantly associated with lower Academic Performance Indices, fewer White students and lower parental education levels. The correlation is significant and positive for the percentage of Black/African American and Hispanic/Latino students as well as those (self-identified) as coming from multi-racial households. The same is true for students who qualify—due to their families’ economic situation—for subsidized or free school lunches. These findings confirm and elaborate on the California-wide results obtained by Green *et al.* [25]. They are also consistent with those of Pastor, Morello-Frosh and Sadd [26] and Mohai *et al.* [27] and Carrier *et al.* [40]. In order to visualize our findings, we subdivided the sample into deciles of the pollution burden variable. The following diagrams (Figure 1) show the mean values of selected socio-economic variables within these deciles.

Figure 1a–d illustrate that the emission loads differ notably between the bottom and top deciles of the PM_2.5_ variable: the mean value in the first decile is 916 times lower than in the 10th, 0.0014 *vs.* 1.2949 g/1000 m^2^ (though the latter also shows the highest standard deviation, the maximum absolute value in this top decile being 3.0136 g/1000 m^2^ or 2152 times the mean value of the first decile). Figure 1a also shows the number of students visiting schools found in each emissions load decile and the relatively sharp changes in the PM_2.5_ means in the top two or three deciles compared to the lower ones. It is interesting to note that the means for both the API2010 and the average parental education level are highest in the third decile. Further analyses would be required, though, to determine whether this effect is an artifact of the way we created the deciles or whether it illustrates a real world phenomenon.

Most of the schools in our sample became operational in the 1980s and like in many other settings see e.g., [41] older, urban schools have experienced growth in the surrounding built environment, which may exacerbate poor environmental conditions. The results do differ to some degree when the sample is subdivided into schools in incorporated and those in unincorporated areas (Table 3), if not along a clear urban/non-urban divide: in the former, no significant correlation was found between PM_2.5_ loads and the percentage of students belonging to two or more races; in the latter no significant relationship was found for the API while one does exist here for percentage of students designated as English learners. The overall picture remains the same throughout, though: schools with higher shares of students belonging to ethnic minorities and/or to socio-economically weaker households tend to be exposed to higher PM_2.5_ loads. 

### 6.2. Environmental Health Screening Variables 

The analysis further showed that our emissions metric is significantly positively correlated with the PM_2.5_, diesel PM and traffic density metrics created for the California Communities Environmental Health Screening Tool [31]. While significance is *p* < 0.005 in all cases, shared variance of the metrics ranges widely. It is highest for *traffic density* in unincorporated areas with R^2^ = 0.286 and lowest for *annual mean PM_2.5_* in incorporated areas with R^2 ^= 0.021 (for this metric, R^2^ is comparatively low across all area types; Table 3). For diesel PM, the comparatively high R^2^ values range between 0.118 and 0.230. 

These findings make sense considering that the on-road component of the diesel PM metric is based on EMFAC (the same source we used for the emissions factors of our calculations but with a more detailed methodology) and modeled transportation networks from local agencies and the California Department of Transportation. The CES traffic density metric on the other hand is based on actual traffic loads on the highway network (taken from the California Department of Transportation’s Highway Performance Monitoring System [31]). The more detailed loaded SACSIM network used to create our PM_2.5_ metric has a larger congruence with the highway network in unincorporated areas than it does in incorporated areas, where it contains a greater share of lower order roads. So the lower shared variance in the latter areas was to be expected. Lastly, the annual mean PM_2.5_ measure from the CES is based on monitoring results and as noted earlier, incorporates sources other than road traffic. Combined with the comparatively small number of monitors in the SACOG region (seven monitoring sites provided continuous data on PM_2.5_ concentrations in 2010 (www.epa.gov/airdata/ad_rep_mon.html) and the resulting coarse resolution of the geostatistically interpolated concentration, the shared variance in the CES metric and that used in our study was expected to be relatively small. The results do show, nevertheless, that while our methodology is the approach with the highest spatial resolution, all related CES measures of air pollution from road traffic we looked at indicate the same spatial trends in emission burdens.

Finally, there was a highly significant association (*p* < 0.005) between a school site’s PM_2.5_ per area load and the rate of emergency department visits for asthma in the tract in which the site is located. The shared variability of the two metrics is comparatively low (*r*^2^ = 0.018) but since firstly the CES metric is not limited to children and secondly, pollution exposure at school is only one among several relevant factors relating to asthma attacks severe enough to require emergency treatment (the only manifestation of the disease captured by the variable), the correlation is still noteworthy. 

### 6.3. Limitations and Use

The method presented here is intended for the comparative assessment of different locations—for example to aid decisions on school siting or the prioritization of measures such as improvements to the heating, ventilation and air-conditioning (HVAC) systems. Actual emissions loads at school sites may vary depending on the vehicle fleet, terrain, time of day and meteorological conditions. We also note that the emission loads, to which a school site is exposed, do not provide a direct indicator of the actual exposure of the students at that school. The latter also depends on the amount and location of indoor *vs.* outdoor activities as well as the quality and use of the HVAC systems. 

It is important to note that our method is new and it would be interesting to see it replicated in other areas. The required data for traffic loads can be taken from travel demand models, which exist for most U.S. metropolitan regions and are widely in use elsewhere (Theoretically, traffic count data could also be used but generally, this is only available for a limited number of road sections in any one region). The input needed from emissions models is also available for a wide geographical coverage (e.g., EMFAC for California, MOVES for the rest of the U.S., HBEFA for Germany, Switzerland and Austria, COPERT for all of Europe). While our method will not yield the type of output required for e.g., the monitoring of ambient air quality standards (which require pollutant measures per volume of ambient air rather than per area) it was developed for relative comparisons of different locations and any generalizations or simplifications stemming from either input data or the method are inherent and will thus not affect the relative assessments. 

## 7. Conclusions

Our study has shown that in public K-12 schools in the SACOG region of California, there is a direct positive correlation between the proportion of Black, Hispanic/Latino and multi-ethnic students as well as students qualifying for subsidized meals and the level of PM_2.5_ from road traffic that the schools are exposed to. At the same time, there is a negative correlation between pollutant levels and the proportion of White students and schools with higher Academic Performance Indices and better educated parent bodies. Furthermore, the pollution loads of the school parcels correlate positively with asthma incidences in the census tracts in which the schools are located. These findings illustrate environmental injustice which is likely to exacerbate health and educational disadvantages that the more affected groups are already experiencing.

Our study also presented a new tool for the site-specific assessment of air quality related to emissions from mobile on-road sources. The results show that our methodology is consistent with the Californian Environmental Protection Agency’s screening measures (CES) of air pollution from road traffic. That is, both measures result in the same spatial trends. However, because our measure is more spatially resolved, down to an actual school parcel, better screening analysis can be performed. The main difference is that current screening tools, like CES, rely on sampling monitors that are limited in number and as a result cannot be used with high confidence to examine specific parcels. Our approach thus provides more proximate estimations of emission loads than both the CES tool [31] and that created by the California Air Resources Board [30]. At the same time it is less resource intensive than the method proposed in school siting guidelines by the U.S. Environmental Protection Agency [20], which makes it suitable for the (comparative) evaluation of a large number of sites.

## Appendix

**Table A1:** Descriptive statistics for pollution burden, schools’ socio-economic characteristics and CES variables (health outcomes, road traffic related pollution indicators).

Analysis Area	Variable	N	Min.	Max.	Mean	SD
ALL	am + md PM_2.5_ (g/1000 m^2^)	510	0	3.014617	0.205991	0.447945
INC.		293	0	3.014617	0.262144	0.537222
UNINC.		217	0	1.983757	0.130171	0.269504
ALL	enrollment	510	2	2450	491	-
INC.		293	13	2450	524	-
UNINC.		217	2	2349	447	-
ALL	Academic Performance Index 2010 (range: 200–1000)	493	402	947	781.2	95.1
INC.		286	402	947	776.8	97.5
UNINC.		207	408	945	787.2	91.4
ALL	% Black/African American Students	510	0	82	9.6	10.53
INC.		293	0	82	10.5	11.55
UNINC.		217	0	43	8.39	8.87
ALL	% Asian Students	510	0	55	8.38	9.19
INC.		293	0	55	10.16	9.85
UNINC.		217	0	37	5.92	7.58
ALL	% Hispanic/Latino Students	510	0	85	28.1	17.53
INC.		293	0	85	31.77	17.71
UNINC.		217	0	82	23.16	16.04
ALL	% White Students	510	0	100	45.12	26.26
INC.		293	0	85	38.31	24.77
UNINC.		217	1	100	54.3	25.44
ALL	% Students of Two or More Races/Ethnicities	510	0	33	3.72	4.03
INC.		293	0	29	3.86	3.98
UNINC.		217	0	33	3.53	4.11
ALL	% Students Eligible for Free/Subsidized Meals	510	0	100	53.41	29.26
INC.		293	0	100	55.11	29.11
UNINC.		217	0	100	51.12	29.37
ALL	% Students Designated as English Learners	510	0	63	16.23	14.89
INC.		293	0	62	17.86	14.76
UNINC.		217	0	63	14.03	14.81
ALL	Average Parental Education Level (1 = No High School to 5 = Grad School)	509	1	4.7	2.94	0.64
INC.		292	1	4.63	2.92	0.67
UNINC.		217	1.75	4.7	2.97	0.61
ALL	CES: Annual Mean PM_2.5_ μg/m^3^	509	5.647	11.433	9.820	1.148
INC.		293	7.181	11.136	9.885	0.974
UNINC.		216	5.647	11.433	9.732	1.345
ALL	CES: Particulate Matter from Diesel (kg/day, July)	510	0.04	34.3	10.72	7.26
INC.		293	0.55	34.3	12.60	7.50
UNINC.		217	0.04	22.22	8.18	6.07
ALL	CES: Traffic Density	506	24	4159	1044	874
INC.		289	24	4159	1115	875
UNINC.		217	52	4135	948	865
ALL	CES: Rate of Asthma Incidences	510	16.10	117.40	51.73	24.94
INC.		293	16.10	117.40	51.10	26.13
UNINC.		217	16.83	117.40	52.58	23.28
ALL	CES: % of Low Weight Births	510	3.95	6.18	5.05	0.43
INC.		293	3.95	6.18	5.07	0.45
UNINC.		217	4.06	6.11	5.03	0.40
